# A novel pN3 gastric cancer staging system with superior prognostic utility based upon the examination of over 31 lymph nodes: a propensity score-matching analysis

**DOI:** 10.1186/s12876-021-01928-w

**Published:** 2021-09-25

**Authors:** Qiantao Hu, Siwei Pan, Zijun Guo

**Affiliations:** 1 Department of Operating Room, Shengjing Hospital of China Medical University, the Sanhao Street 36, Shenyang, 110001 China; 2 Department of Surgical Oncology, First Hospital of China Medical University, Shenyang, China

**Keywords:** Gastric cancer, pN3 stage, Examined lymph node, Metastatic lymph node, Propensity score-matched analysis

## Abstract

**Background:**

Individuals with pN3 gastric cancer (GC) account for a large proportion of pN + GC, and exhibit poor survival outcomes. The pN3 stage is defined based upon the number of metastatic lymph nodes (mLNs), but the subclassification of pN3 patients based upon the number of examined LNs (eLNs) is rarely performed.

**Methods:**

In total, 2894 pTxN3M0 GC patients in the Surveillance, Epidemiology, and End Results database that had undergone surgery from 2000 to 2016 were selected for analysis. The X-tile software was used to select the optimal cutoff values. Cox proportional regression analyses were used to evaluated hazard ratios corresponding to the risk of death. Selection bias was minimized via propensity score matching (PSM).

**Results:**

As the number of eLNs rose, the risk of death for patients trended downwards. Survival analyses indicated that patients with ≤ 31 eLNs exhibited significantly poorer survival outcomes as compared to patients with > 31 eLNs (5-year OS: 18.4% vs. 24.7%), and this result remained significant when analyzing 857 pairs of patients following PSM analysis. Significant differences in prognosis were additionally observed when comparing pN3a and pN3b patients with ≤ 31 or > 31 eLNs under pT3/4a stage. For pT4b stage, pN3a patients with > 31 eLNs also exhibited a better prognosis than other patients. The novel TNM staging system designed exhibited excellent utility as a tool for the prognostic evaluation of this GC patient population.

**Conclusions:**

These results suggest that in pN3 GC, a minimum of 32 LNs should be examined. The novel TNM staging system for pN3 patients described herein, which was developed based upon the number of eLNs, may thus be of value in clinical settings.

## Background

Gastric cancer (GC) is the fifth most common and fourth deadliest cancer type, with over 1 million diagnoses and 760,000 deaths in 2020 alone [1]. While GC patient 5-year survival rates have been slowly rising in China, they remain under 35.1% for all GC patients and under 10% for those with advanced disease [2, 3]. Surgical tumor resection remains the primary treatment for advanced GC [4, 5], but accurately assessing and staging GC patients is valuable as a means of guiding clinical decision-making.

The Union for International Cancer Control/American Joint Committee on Cancer (UICC/AJCC) staging system is an internationally accepted set of criteria that is widely used in clinical practice [6, 7]. Under these guidelines, the N stage assesses the degree of lymph node metastasis based on the number of metastatic lymph nodes (mLNs), yet it fails to take into account the number of examined lymph nodes (eLNs). Patients with ≥ 7 mLNs in resected samples are diagnosed with pN3 stage disease. In Chinese, Japanese, Korean, and Western GC patient cohorts, these patients account for 39.2–50%, 36.2%, 40.2%, and 42.1% of all patients with mLNs, respectively [8–12]. Many studies have focused on subtyping patients with pN3b stage disease based upon the number of identified mLNs [13, 14]. While National Comprehensive Cancer Network (NCCN) guidelines recommend that a minimum of 15 lymph nodes be examined in GC patients to reduce staging migration [15], this number is not sufficient for patients with ≥ 7 mLNs diagnosed with pN3 stage disease, particularly for pN3b stage patients with ≥ 16 mLNs. Multiple prior analyses have revealed that for patients with certain TNM stages of disease, the optimal number of eLNs associated with improved patient prognosis may be 23 or higher [9, 11, 16, 17]. There is thus a clear need to further refine the definitions of pN3 patient subclassifications in order to further optimize the AJCC-TNM staging system.

As such, in light of the AJCC-TNM staging system, related guidelines, and other research results, this study was formulated to further subclassify pN3 stage GC patients, who make up a large proportion of GC patients, based upon numbers of mLNs and eLNs using the Surveillance, Epidemiology, and End Results (SEER) database.

## Methods

### Study population

**Fig. 1:**
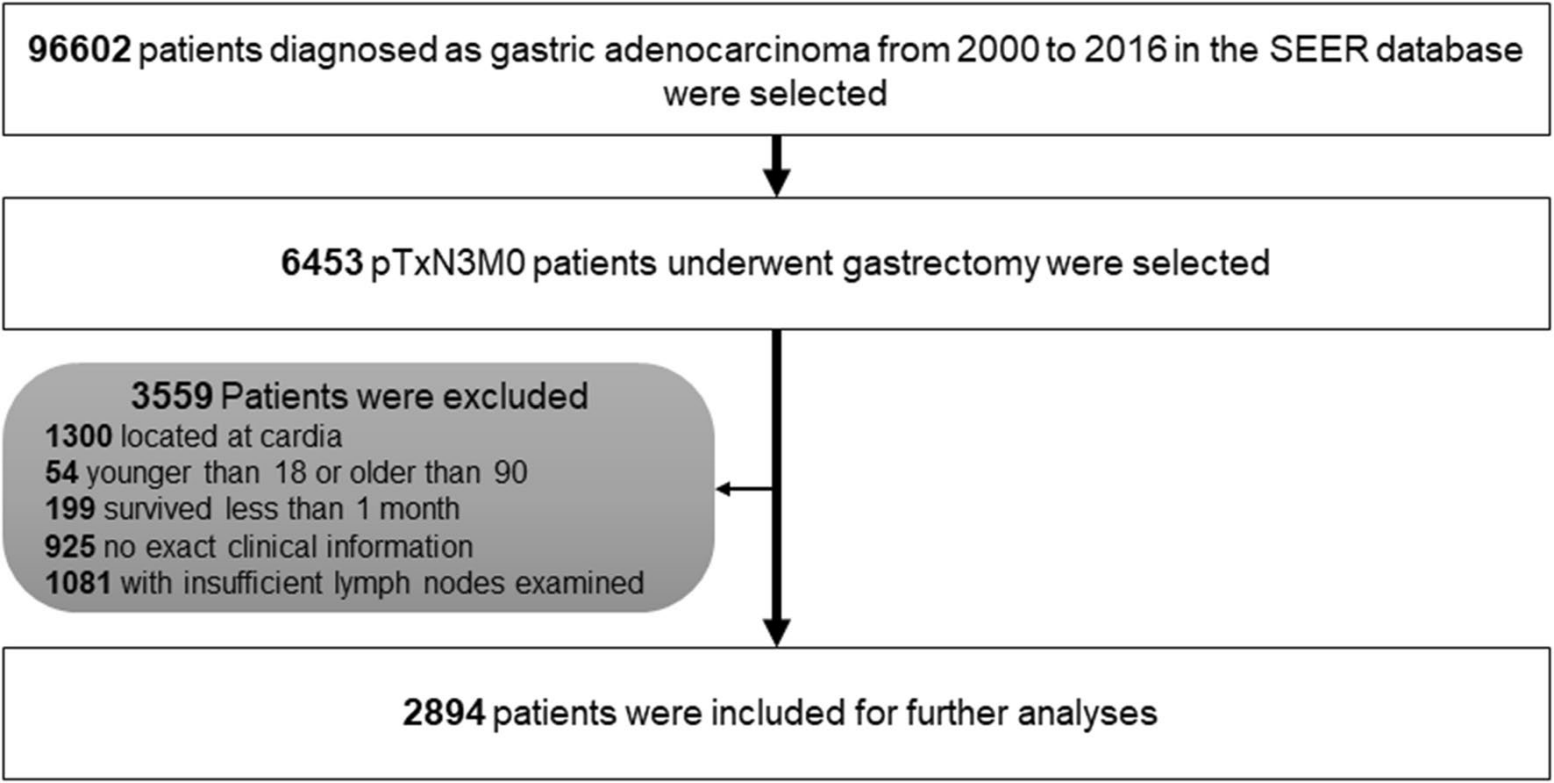
Patient screening process for the current study from the SEER database

The SEER program compiles authoritative cancer incidence and survival data pertaining to roughly 28% of the US population [18, 19]. The SEER-STAT software (SEER*Stat 8.3.6) was employed to screen the data for the present study. Patients that had undergone gastrectomy who were subsequently diagnosed with gastric adenocarcinoma between 2000 and 2016 who were included in the SEER database were identified. Patients with > 6 mLNs (pN3) and without distant metastases (pM0) as per the 8^th^ edition AJCC Cancer Staging Manual were selected for further analysis. Patients were excluded from this study if they: (1) exhibited tumors at the cardia; (2) were < 18 or > 90 years old; (3) lacked clear follow-up or clinical data; (4) survived for < 1 month; (5) had < 16 eLNs. Based upon these criteria, 2894 patients were eligible for inclusion in this study (Fig. 1).

Clinicopathological characteristics extracted from the SEER database included age at diagnosis, race, sex, tumor grade, tumor primary site, tumor size, tumor depth of invasion, number of eLNs, number of mLNs, adjuvant therapy, and patient outcomes as of most recent follow-up (Nov. 2018). TNM staging was defined according to the 8th edition of the AJCC Staging Manual.

### Propensity score-matching (PSM) analysis

As the SEER program did not record patient data based upon the number of eLNs in a random manner, subgroup analyses would be subject to intrinsic bias. To minimize the potential impact of such selection bias and associated confounding variables when separating patients into groups, a PSM analysis was thus conducted [20, 21]. A 1:1 matching approach without replacement was performed using a nearest-neighbor matching based upon the logit of the propensity score within a caliper of 0.01, with this score having been derived based on sex, age, grade, primary site, tumor size, T stage, N stage, and adjuvant therapy type.

### Statistical analysis

Categorical variables are given as counts and proportions, and were analyzed using Pearson’s Chi-squared tests or Fisher’s exact test. Overall survival (OS) was defined as the time between tumor resection and death, and served as a key composite prognostic readout. OS values in different patient groups were compared using Kaplan–Meier curves and log-rank tests, with follow-up being quantified via the reverse Kaplan–Meier method [22]. The X-tile software (https://medicine.yale.edu/lab/rimm/research/software.aspx) was utilized to select the optimal eLN cutoff value for the reliable classification of pN3 patients so as to maximize prognostic accuracy [23]. Cox proportional hazards regression models were employed to establish hazard ratios (HRs) for prognostic variables of interest, with Cox proportional regression analyses with a restricted cubic spline model being conducted to examine relationships between continuous variables and HRs [24]. Time-dependent receiver operating characteristic (ROC) curves were generated, and the area under the curve (AUC) was measured to gauge the accuracy of a given classification. To measure clinical utility, a decision curve analysis (DCA) was conducted by measuring the net benefits for a group of threshold probabilities. Likelihood ratio χ^2^ tests were used to assess homogeneity within a given classification, with the linear trend χ^2^ test was used to assess discriminatory ability and gradient monotonicity (for patients with favorable clinical features exhibiting prolonged survival relative to those with unfavorable conditions). The discriminatory ability of each classification was evaluated using Akaike information criterion (AIC) and Bayesian information criterion (BIC) values, with smaller AIC and BIC values being indicative of better prognostic utility [25].

R (v 3.6.0; R Foundation for Statistical Computing, Vienna, Austria) and SPSS (v 23.0; SPSS Inc, IL, USA) were used to conduct all statistical analyses, with *P* < 0.05 as the significance threshold.

## Results

### GC patient clinicopathological characteristics

The clinicopathological characteristics of the 2894 patients with pN3 stage GC identified in the SEER database who were eligible for inclusion in the present study are compiled in Table 1. The median age of these patients at the time of diagnosis was 67 years, and a majority of these patients were male (1661, 57.4%), with more than one-third having been diagnosed with GC affecting the lower third of the stomach (1081, 37.4%). The median tumor size for this patient cohort was 6 cm, with 1383 (47.8%) patients exhibiting a tumor > 6 cm in size. Approximately 60 percent of patients were diagnosed with pN3a stage disease (1763, 60.9%). The mean numbers of eLNs and mLNs in these patients were 29.05 ± 13.44 and 15.65 ± 8.48, respectively. Approximately two-thirds of patients (1940, 67.0%) underwent postoperative adjuvant therapy. The median follow-up time for these patients was 93 months (range: 0 – 203), and their 5-year OS was 20.10%.

**Table 1:** Basic clinicopathological characteristics of the 2894 patients with pN3 stage GC

Characteristic	All Patients (n = 2894)	
	n	%
*Age*		
≤ 60	1009	34.9
> 60	1885	65.1
*Race*		
White	1659	57.3
Black/Others*	1235	42.7
*Sex*		
Male	1661	57.4
Female	1233	42.6
*Primary site*		
Upper	115	4.0
Middle	358	12.4
Lower	1081	37.4
Curvature	668	23.1
Overlapping lesion	398	13.8
Stomach, NOS	274	9.5
*Grade*		
Well differentiated	18	0.6
Moderately differentiated	406	14.0
Poorly differentiated	2388	82.5
Undifferentiated	82	2.8
*Size*		
≤ 6 cm	1511	52.2
> 6 cm	1383	47.8
*pT stage*		
pT1a	10	0.3
pT1b	59	2.0
pT2	152	4.3
pT3	1220	42.2
pT4a	1102	38.1
pT4b	351	12.1
*pN stage*		
pN3a	1763	60.9
pN3b	1131	39.1
*TNM stage*		
IIB	55	1.9
IIIA	118	4.1
IIIB	1447	50.0
IIIC	1274	44.0
*Adjuvant therapy*		
Observation	954	33.0
ART	83	2.9
ACT	664	22.9
ACRT	1193	41.2

n, number of patients; NOS, not otherwise specified; ART, adjuvant radiotherapy; ACT, adjuvant chemotherapy; ACRT, adjuvant chemoradiotherapy

*Referring to American Indian/AK Native, Asian/Pacific Islander

### Assessment of the prognostic relevance of eLNs and mLNs

**Fig. 2:**
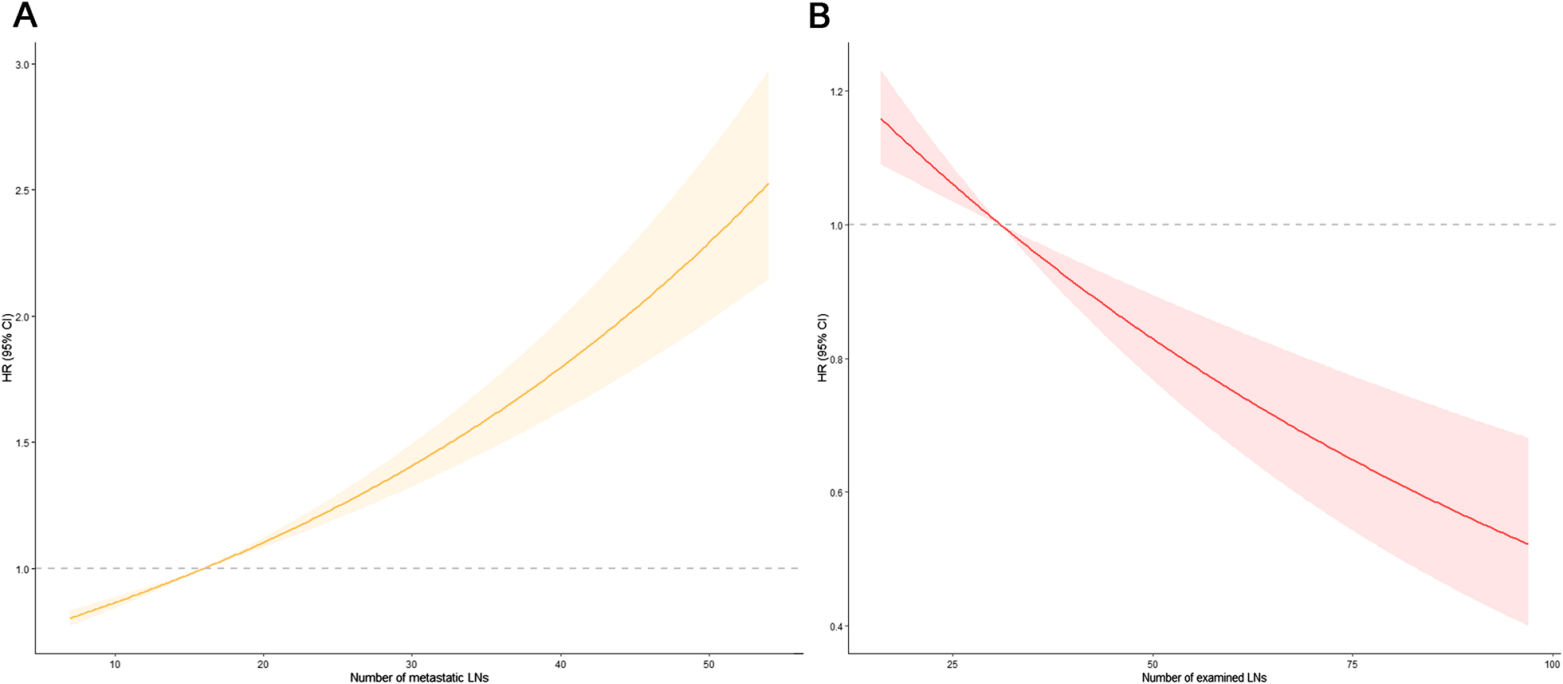
The association between the number of mLNs (**A**), eLNs (**B**) and HRs for pN3b patients by using the univariate Cox proportional regression analyses with a restricted cubic spline model

To examine the relationship between the number of mLNs or eLNs and GC patient mortality risk, we conducted Cox proportional regression analyses using a restricted cubic spline model. HRs rose significantly as the number of mLNs increased (Fig. 2A), with higher numbers of mLNs being associated with an increased risk of death. In contrast, HRs declined rapidly as the number of eLNs increased (Fig. 2B), suggesting that GC patient survival outcomes differ significantly in a manner correlated with the number of eLNs.

### Cut‑off value selection, PSM, and survival analyses

**Fig. 3:**
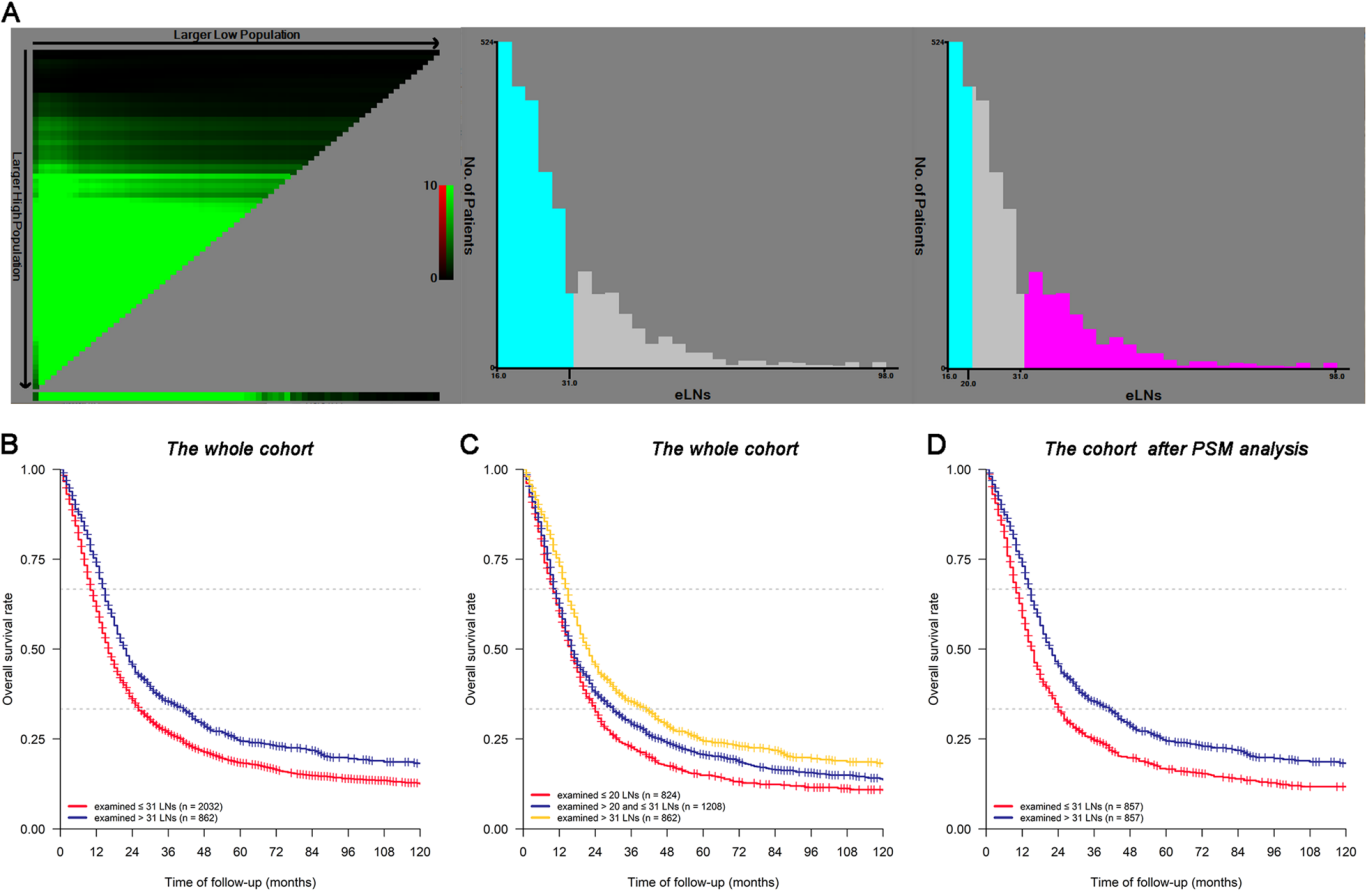
Calculation of the pN3 patients using the optimal obtained cut-off values of eLNs using the X-tile software (**A**). Survival curves of the pN3 patients using the optimal cut-off values of eLNs: **B** patients with ≤ 31 eLNs vs. patients with > 31 eLNs; **C** patients with ≤ 20 eLNs vs. patients with > 20 and ≤ 31 eLNs vs. patients with > 31 eLNs; **D** patients with ≤ 31 eLNs vs. patients with > 31 eLNs after PSM analysis

In light of the apparent relationship between eLNs and HRs in pN3 GC patients detected above, we next sought to use the X-tile software to establish an optimal eLN cutoff value capable of maximizing prognostic accuracy when evaluating these patients. Prior to PSM analyses, the optimal number of eLNs for separating 2894 patients into two categories was 31, while the best cutoff values for three categories were 20 and 31 (Fig. 3A). Survival analyses indicated that patients with ≤ 31 eLNs exhibited significantly worse survival outcomes relative to patients with > 31 eLNs (5-year OS: 18.4% vs. 24.7%, *P* < 0.001, Fig. 3B). Significant differences in survival outcomes were also observed among groups when separated into three categories according to the cutoff values of 20 and 31 eLNs (5-year OS: 14.9% vs. 20.7% vs. 24.4%, *P* < 0.001, Fig. 3C). In order to facilitate clinical decision-making, we separated patients into two groups based upon the number of eLNs 31 (≤ 31 or > 31) and conducted a PSM analysis. Following this analysis, 857 pairs of pN3 stage GC patients with ≤ 31 or > 31 eLNs remained, thereby minimizing the potential impacts of confounding variables and selection bias on analytical results (Table 2, All *P* > 0.05 after matching). Even after such matching, patients with > 31 eLNs exhibited a 5-year OS that was almost 8% higher than that observed for patients with ≤ 31 eLNs (5-year OS: 16.6% vs. 24.4%, *P* < 0.001, Fig. 3D).

**Table 2:** Clinicopathological characteristics of patients grouped by the optimal cut-off value of eLNs before and after PSM analysis

Characteristic	Before PSM analysis					After PSM analysis				
	Examined ≤ 31 LNs (n = 2032)		Examined > 31 LNs (n = 862)		*P* value	Examined ≤ 31 LNs (n = 857)		Examined > 31 LNs (n = 857)		*P* value
	n	%	n	%		n	%	n	%	
*Age*					0.097					0.920
≤ 60	689	33.9	320	37.1		317	37.0	315	36.8	
> 60	1343	66.1	542	62.9		540	63.0	542	63.2	
*Race*					0.213					0.143
White	1180	58.1	479	55.6		507	59.2	477	55.7	
Black/Others*	852	41.9	383	44.4		350	40.8	380	44.3	
*Sex*					0.758					0.128
Male	1170	57.6	491	57.0		519	60.6	488	56.9	
Female	862	42.4	371	43.0		338	39.4	369	43.1	
*Primary site*					0.164					0.806
Upper	80	3.9	35	4.1		35	4.1	35	4.1	
Middle	235	11.6	123	14.3		112	13.1	121	14.1	
Lower	786	38.7	295	34.2		320	37.3	294	34.3	
Curvature	461	22.7	207	24.0		188	21.9	206	24.0	
Overlapping lesion	274	13.5	124	14.4		122	14.2	123	14.4	
Stomach, NOS	196	9.6	78	9.1		80	9.3	78	9.1	
*Grade*					0.053					0.154
Well differentiated	14	0.7	4	0.5		7	0.8	4	0.5	
Moderately differentiated	295	41.5	111	12.9		119	13.9	108	12.6	
Poorly differentiated	1656	81.5	732	84.9		704	82.1	730	85.2	
Undifferentiated	67	3.3	15	1.7		27	3.2	15	1.8	
*Size*					**0.006**					0.074
≤ 6 cm	1095	53.9	416	48.3		451	52.6	414	48.3	
> 6 cm	937	46.1	446	51.7		406	47.4	443	51.7	
*pT stage*					0.394					0.441
pT1a	7	0.3	3	0.4		4	0.5	3	0.4	
pT1b	39	1.9	20	2.3		19	2.2	20	2.3	
pT2	109	5.4	43	5.0		45	5.3	41	4.8	
pT3	866	42.6	354	41.1		373	43.5	352	41.1	
pT4a	752	37.0	350	40.6		308	35.9	349	40.7	
pT4b	259	12.7	92	10.7		108	12.6	92	10.7	
*pN stage*					** < 0.001**					0.923
pN3a	1380	67.9	383	44.4		381	44.5	383	44.7	
pN3b	652	32.1	479	55.6		476	55.5	474	55.3	
*TNM stage*					** < 0.001**					0.951
IIB	39	1.9	16	1.9		19	2.2	16	1.9	
IIIA	90	4.4	28	3.3		28	3.3	28	3.3	
IIIB	1118	55.0	329	38.2		320	37.3	327	38.2	
IIIC	785	38.6	489	56.7		490	57.2	486	56.7	
*Adjuvant therapy*					** < 0.001**					0.064
Observation	710	34.9	244	28.3		265	30.9	244	28.5	
ART	56	2.8	27	3.1		23	2.7	27	3.2	
ACT	426	21.0	238	27.6		192	22.4	238	27.8	
ACRT	840	41.3	353	41.0		377	44.0	348	40.6	

Bold values indicate the significant difference with *P* < 0.05

n, number of patients; LNs, lymph nodes; PSM, propensity score matching; NOS, not otherwise specified; ART, adjuvant radiotherapy; ACT, adjuvant chemotherapy; ACRT, adjuvant chemoradiotherapy

*Referring to American Indian/AK Native, Asian/Pacific Islander

### Subgroup survival comparisons for patients with different numbers of eLNs

**Fig. 4:**
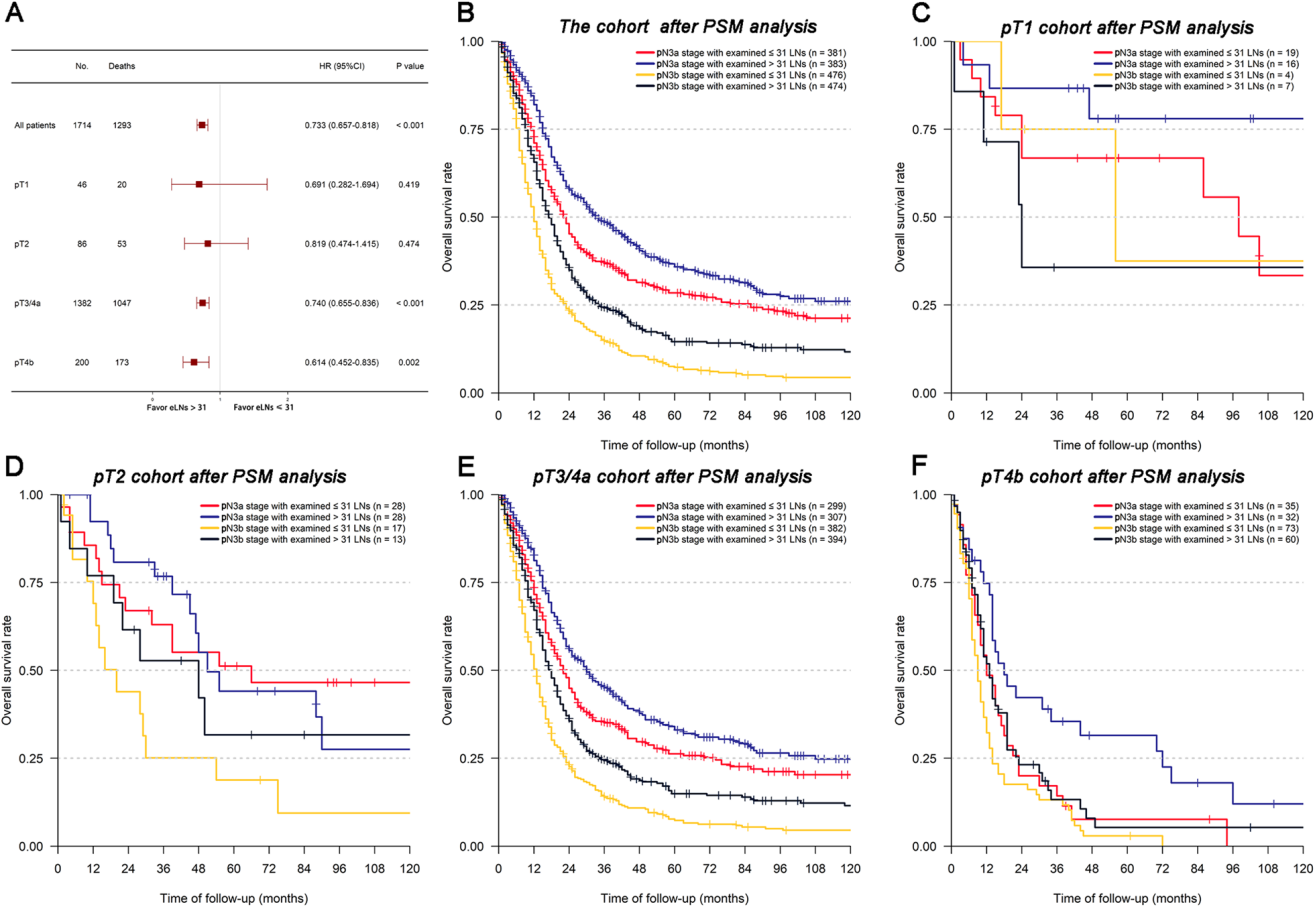
Subgroup survival analyses and forest plot of pN3 patients under different pT stages after PSM analysis: **A** forest plot of HRs and 95% CIs for OS of patients examined ≤ 31 or > 31 LNs; **B** survival curves of the whole matched pN3 cohort; **C** survival curves of pT1 stage cohort; **D** survival curves of pT2 stage cohort; **E** survival curves of pT3/4a stage cohort; **F** survival curves of pT4b stage cohort

As shown in Fig. 4A, we found that the prognosis of pN3 patients with ≤ 31 or > 31 eLNs differed among different pT stages. For pT1 or pT2 patients, although lower HRs were evident for individuals with > 31 eLNs (HR = 0.691 and 0.819, respectively), there were no significant differences in survival when comparing individuals with ≤ 31 or > 31 eLNs (All *P* > 0.05). Conversely, patients with > 31 eLNs exhibited a significantly better prognosis than those with ≤ 31 eLNs for pT3/4a and pT4b stages (pT3/4a, HR = 0.740, *P* < 0.001; pT4b, HR = 0.614, *P* = 0.002). We next separated all pN3a and pN3b stage GC patients into four groups according to the number of eLNs: pN3a patients with ≤ 31 or > 31 eLNs, and pN3b patients with ≤ 31 or > 31 eLNs. As shown in Fig. 4B, these four groups exhibited significantly different prognoses (All *P* < 0.05), with pN3b patients with ≤ 31 eLNs having the worst prognosis (5-year OS: 7.3%). For patients with a given pN stage, those with > 31 eLNs exhibited better survival outcomes than those with ≤ 31 eLNs (pN3a stage, 5-year OS: 35.9% vs. 28.5%, *P* = 0.004; pN3b stage, 5-year OS: 14.6% vs. 7.3% *P* < 0.001). We additionally conducted subgroup analyses of pN3 patients with different pT stages. In pT1 stage patients, no significant differences in survival outcomes were observed among groups, likely due to the small number of patients in this cohort (All *P* > 0.05, Fig. 4C). In pN3a or pN3b patients in the pT2 cohort, there were also no significant differences between patients with ≤ 31 or > 31 eLNs (Fig. 4D). However, pN3b patients with ≤ 31 eLNs exhibited a significantly worse prognosis than both pN3a patient groups (pN3a patients with ≤ 31 eLNs, *P* = 0.016; pN3a patients with > 31 eLNs, *P* = 0.009). In the cohort of patients with pT3/4a stage disease, there were significant differences in survival outcomes among these four groups (All *P* < 0.05, Fig. 4E). Patients with pT4b stage disease additionally exhibited significant differences in survival among these four groups (Fig. 4F), with pN3a patients exhibiting a significantly better prognosis (5-year OS: 31.5%, *P* < 0.05), whereas there were no significant survival differences among the other three groups (pN3a patients with ≤ 31 eLNs, 5-year OS: 7.6%; pN3b patients with ≤ 31 eLNs, 5-year OS: 2.9%; pN3b patients with > 31 eLNs, 5-year OS: 5.3%).

### Establishment and evaluation of a novel TNM staging system for pN3 stage GC patients

**Fig. 5:**
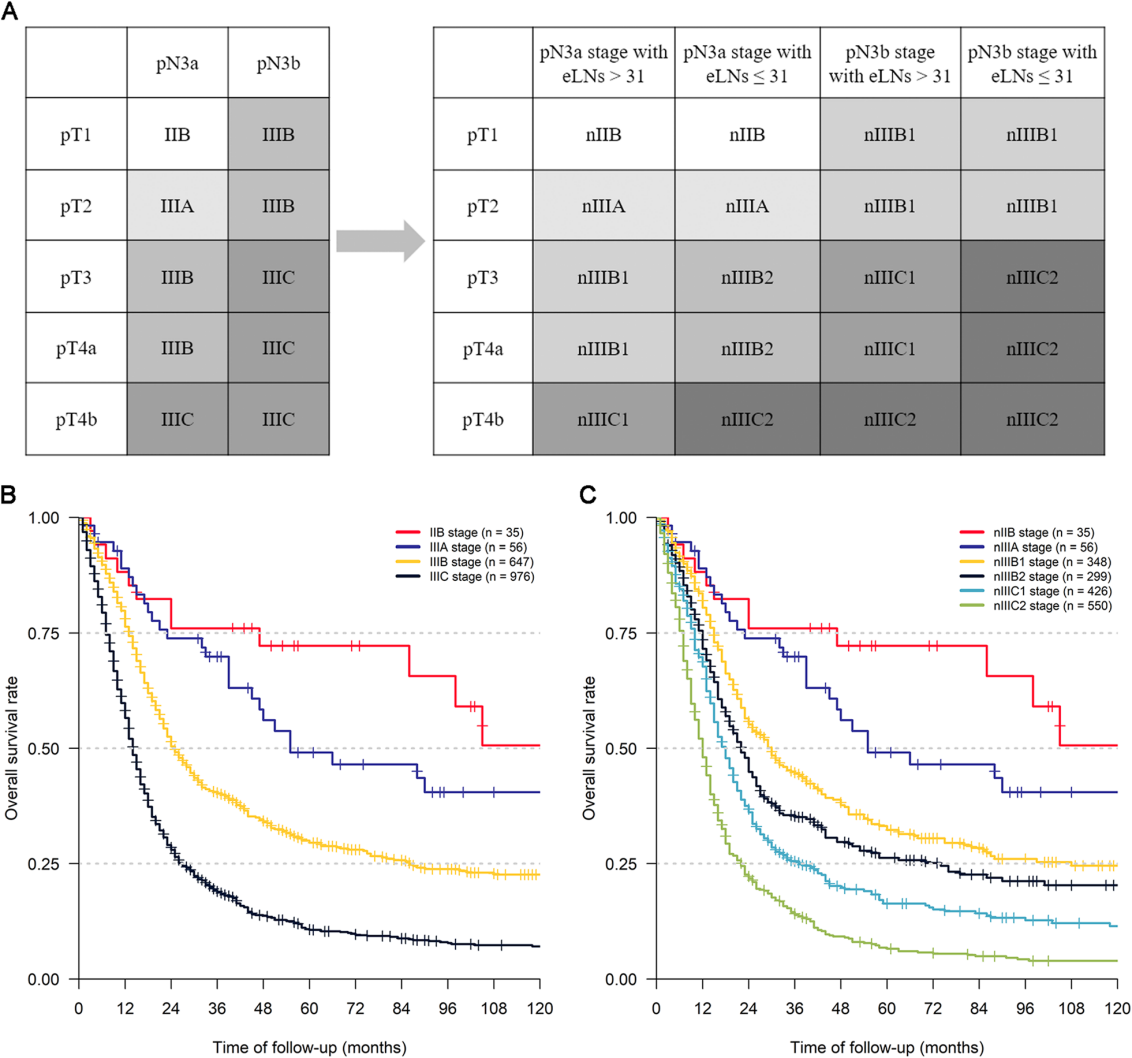
The novel TNM staging system for pN3 stage GC patients taking the number of eLNs into account were established (**A**). Survival curves of pN3 patients under different staging systems: **B** AJCC-TNM staging system; **C** the novel TNM staging system. ROC curves of pN3 patients under different staging systems for predicting OS

**Fig. 6:**
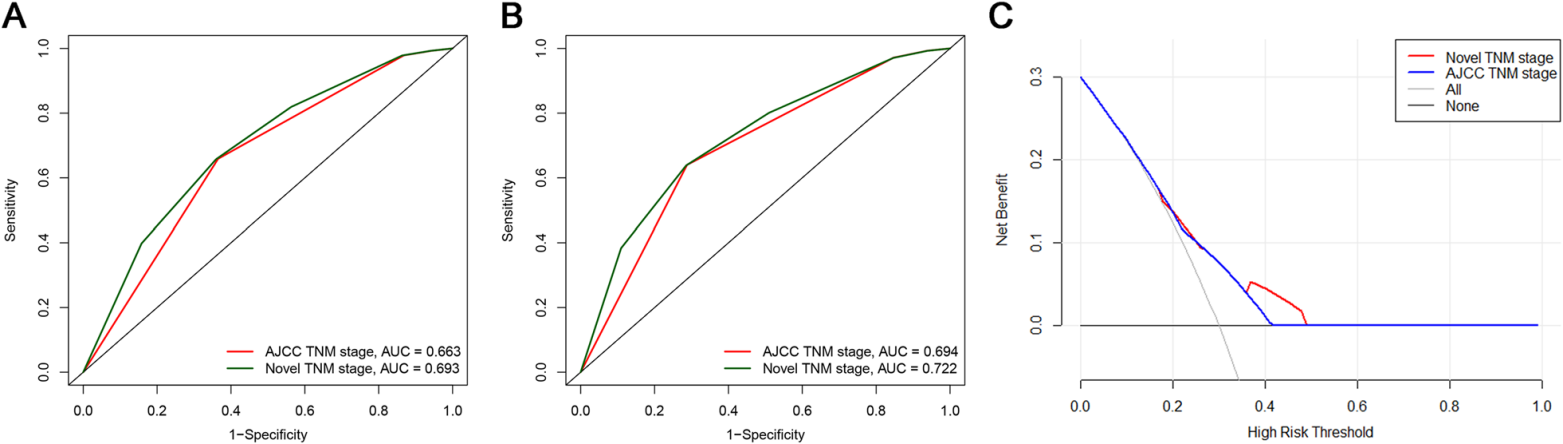
ROC curves of pN3 patients under different staging systems for predicting OS: **A** 3-year OS; **B** 5-year OS. **C** The DCA curves of pN3 patients under AJCC-TNM and nTNM staging systems

In light of our above subgroup analyses, we modified the AJCC-TNM staging system for pN3 GC patients and proposed a novel TNM (nTNM) staging system that takes the number of eLNs into account (Fig. 5A). In this nTNM staging system, pN3 patients were separated into six groups with distinct prognoses. For those patients with pT3 or higher stage disease, the classification system was expanded from the original two classifications to four under our nTNM staging system. Survival curves for the AJCC-TNM and nTNM staging systems are shown in Fig. 5B and C. While both systems were able to effectively classify pN3 patients according to their survival outcomes, our novel system was more precise as a classification tool. When the 3-year OS of pN3 patients was assessed, the AUC values for the AJCC-TNM and nTNM staging systems were 0.669 and 0.693, respectively (Fig. 6A), while for 5-year OS these values were 0.694 and 0.722, respectively (Fig. 6B). DCA curves also revealed that the nTNM staging system exhibited better clinical utility when used for prognostic analyses as compared to the AJCC TNM staging system (Fig. 6C). The homogeneity, discriminatory ability, and monotonicity of gradients were improved for this nTNM staging system, with higher linear trend χ^2^ and likelihood ratio χ^2^ values relative to those associated with AJCC-TNM staging (Table 3). Furthermore, the smaller AIC and BIC values associated with our novel system suggested that it may be an optimal tool for prognostic patient stratification.

**Table 3:** Comparison of the performance of the AJCC-TNM staging system and the novel TNM staging system

	Liner trend χ^2^	Likelihood ratio χ^2^	AIC	BIC
AJCC-TNM	85.05 (*P* < 0.001)^*^	81.02 (*P* < 0.001)^#^	17,089.70	17,094.87
nTNM	103.63 (*P* < 0.001)^*^	108.67 (*P* < 0.001)^#^	17,048.93	17,054.1

*Comparison of overall survival by liner trend χ2 test among different stages

^#^Comparison of overall survival by likelihood ratio χ2 test among different stages

## Discussion

Herein, we examined the prognostic relevance of different numbers of eLNs in 2894 pTxN3M0 GC patients in the SEER database who had undergone gastrectomy. Following PSM analyses aimed at controlling for selection bias and confounding variables, 857 patient pairs were retained for subsequent analyses which indicated that pN3 GC patients with > 31 eLNs survived for longer than did individuals with fewer eLNs. Based on these results, we proposed an optimized version of the AJCC-TNM staging system for these patients, and found that this nTNM staging system was more reliably able to predict patient prognosis as compared to the 8^th^ edition AJCC-TNM staging system.

Currently, pN3 stage GC is pathologically diagnosed based upon the identification of 7 or more mLNs in postoperative tissue specimens, with a cutoff of 16 mLNs being used to further stratify these patients into those with pN3a and pN3b stage disease [6]. An estimated 15.7% of total GC patients in the world are diagnosed with pN3 stage disease, accounting for 38.1% of patients with pN + disease [12]. Among patients free of distant metastases other than those with pT1N3aM0 early-stage GC, which is classified as stage IIB under the AJCC-TNM staging system, all other pN3 patients are classified as having stage III disease [6, 7]. A single-center retrospective analysis conducted in China determined that among M0 stage patients with matched T stage disease, pN3 stage patients exhibited a worse prognosis than other patients, with a 5-year OS as low as 10.5% (pT4bN3bM0) or 7.1% (pT4aN3bM0), whereas the 5-year OS for M1 patients was 7.6% [8]. Similarly, pN3a GC has been linked to a poor prognosis in Western patient cohorts, with pN3a stage disease being associated with a 5-year OS of approximately 20%, falling to under 10% in those with pN3b stage disease [26]. While pN3 patients generally exhibit a poor prognosis, the 5-year OS of these patients varies significantly from 7.1 to 62.5% across different TNM stages [8].

AJCC-N staging according to the number of identified mLNs has been confirmed to be a key prognostic indicator in multiple multicenter retrospective analyses of Chinese, Western, and global populations [12, 26, 27]. Several studies have, to date, sought to further optimize such AJCC-N staging based upon the number of mLNs and/or eLNs [9, 11, 13, 14, 28–31]. For example, one multicenter Chinese cohort study led to the proposal of a novel mLN number-based subclassification system for pN3b GC patients [15]. Specifically, pN3b patients with > 24 mLNs were found to exhibit a significantly lower 5-year OS relative to patients with 16–24 mLNs (13.5% vs. 16.4%, *P* = 0.048). Another single-center study of 222 GC patients proposed a cutoff value of 21 for further stratifying such pN3b patients [16]. With respect to the number of eLNs, one 10-year retrospective study found that pN2-N3 patients in whom at least 25 LNs had been evaluated exhibited a better prognosis than did other patients, exhibiting a roughly 10% improvement in their 5-year OS among pN3 patients [11]. Zheng et al. [9] detected no significant differences in pN1 or pN2 patient prognosis as a function of the number of eLNs, whereas they found that the examination of > 22 LNs was linked to significantly prolonged survival following radical gastrectomy. Herein, we also determined that pN3 stage GC patients with > 31 eLNs exhibited a better prognosis than those with fewer eLNs both before and after PSM analysis (All *P* < 0.001). Given that pN3 patients make up a large fraction of total GC patients and have an inconsistent prognosis, we suggest that it is important to subclassify these patients not only based upon the number of mLNs, but also on the number of eLNs, leading us to propose a new staging system. Indeed, the LN ratio (LNR) and log-odds of metastatic lymph nodes (LODDs) staging systems that take both mLN and eLN numbers into account have been validated in multiple previous reports [28–31]. While promising, the implementation of these two prior systems was complicated, making them impractical for use in clinical practice.

In addition, although several studies have suggested that more LNs should be examined in pN3 patients [9, 11] and the main subjects of the present study were pN3 GC patients with sufficient eLNs, it remains challenging to examine a sufficient number of LNs in certain clinical contexts, such as in patients after neoadjuvant or conversion therapy, or as a consequence of limited clinician experience in performing LN examinations. For these patients, we posit that eLN-based staging optimization should also be performed. The results of one retrospective study combining data from multiple centers and the SEER database found that pN3a patients with < 16 eLNs exhibited a significantly poorer prognosis relative to patients with ≥ 16 eLNs, and should thus be classified as having pN3b stage disease [32]. In another single-center study focused on patients with stage III GC, researchers found that at stages IIIA, IIIB, and IIIC, the prognosis of patients with < 16 eLNs was significantly poorer than that of patients with ≥ 16 eLNs, suggesting that there were large substage increases in stage III patients with insufficient eLNs [33].In the present study, following PSM analyses conducted to control for potential confounding factors and selection bias, we confirmed that pN3 patients with > 31 or ≤ 31 eLNs still exhibited significantly different prognoses within the SEER cohort. However, due to sample size limitations we were unable to detect significant differences in survival outcomes as a function of the number of eLNs in pN3a or pN3b patients with pT1 or pT2 stage disease, although we did detect significant differences in pT3 and pT4 stage patient outcomes such that we were able to divide this population into four subgroups rather than the original two AJCC-TNM stages (Fig. 5A). We further confirmed that our novel staging system offered prognostic advantages over the AJCC-TNM staging system when evaluating pN3 patients.

Despite our promising results, this study is subject to certain limitations. For one, this was a retrospective analysis of the SEER database, which compiles data from many centers over an extended period of time, potentially introducing variability with respect to patient diagnosis and treatment strategies. In addition, our pT1/2 patient sample size was limited, reducing our statistical power when conducting prognostic analyses of these patients. Furthermore, while a PSM approach was employed to decrease the influence of bias on our study results, this approach is not comparable to the data generated by a randomized control study. Future prospective randomized controlled studies and/or larger retrospective analyses are thus warranted to validate and expand upon our results. The AUC improvements observed for ROC curve analyses and the changes in AIC and BIC indexes associated with this novel TNM staging system were limited, potentially owing to sample size limitations and biases associated with different treatment regimens across centers in the SEER database. However, the primary significance of the present study was not only that we were able to further optimize the AJCC TNM staging system, but also that we were able to provide evidence suggesting that there were certain limitations associated with staging based solely on the number of mLNs for pN3 GC patients. More detailed staging systems based upon the numbers of both eLNs and mLNs may thus represent a valuable future direction for the precision medicine-based treatment of GC.

## Conclusions

In summary, we were able to further subclassify patients with pN3 stage GC using an optimal eLN cutoff number of 31 identified using the SEER database. Patients attained a significant survival benefit in the present study if they underwent the examination of > 31 LNs. Subgroup-based analyses of pT stages further revealed that there were significant differences in the prognostic outcomes of pN3a/b stage patients with > 31 eLNs relative to those of patients with ≤ 31 eLNs. In light of these analyses, we additionally proposed a novel TNM staging system capable of differentiating pN3 patients into six prognosis-related subgroups. Future external prospective studies will be essential to validate the utility of this new TNM staging approach.

## Data Availability

The datasets generated and analyzed during the current study are available in the SEER database (https://seer.cancer.gov/) and from the corresponding authors upon reasonable request.

## Abbreviations

GC: Gastric cancer
mLNs: Metastatic lymph nodes
eLNs: Examined lymph nodes
PSM: Propensity score matching
UICC/AJCC: Union for International Cancer Control/American Joint Committee on Cancer
NCCN: National Comprehensive Cancer Network
SEER: Surveillance, Epidemiology, and End Results
OS: Overall survival
ROC: Receiver operating characteristic
AUC: Area under the curve
DCA: Decision curve analysis
AIC: Akaike information criterion
BIC: Bayesian information criterion
nTNM: Novel TNM
LNR: Lymph node ratio
LODDs: Log-odds of metastatic lymph nodes

## References

[1] Sung H, Ferlay J, Siegel RL (2021). Global cancer statistics 2020: GLOBOCAN estimates of incidence and mortality worldwide for 36 cancers in 185 countries. CA Cancer J Clin.

[2] Zeng H, Chen W, Zheng R (2018). Changing cancer survival in China during 2003–15: a pooled analysis of 17 population-based cancer registries. Lancet Glob Health.

[3] Yang L, Ying X, Liu S (2020). Gastric cancer: Epidemiology, risk factors and prevention strategies. Chin J Cancer Res.

[4] Feng RM, Zong YN, Cao SM, Xu RH (2019). Current cancer situation in China: good or bad news from the 2018 Global Cancer Statistics?. Cancer Commun (Lond).

[5] Joshi SS, Badgwell BD (2021). Current treatment and recent progress in gastric cancer. CA Cancer J Clin.

[6] Washington K (2010). 7th edition of the AJCC cancer staging manual: stomach. Ann Surg Oncol.

[7] Sobin LH, Wittekind CH (1997). TNM classification of malignant tumors (UICC).

[8] Sun Z, Wang ZN, Zhu Z (2012). Evaluation of the seventh edition of American Joint Committee on Cancer TNM staging system for gastric cancer: results from a Chinese monoinstitutional study. Ann Surg Oncol.

[9] Zheng G, Feng F, Guo M (2017). Harvest of at least 23 lymph nodes is indispensable for stage N3 gastric cancer patients. Ann Surg Oncol.

[10] Deng J, Zhang R, Pan Y (2014). Comparison of the staging of regional lymph nodes using the sixth and seventh editions of the tumor-node-metastasis (TNM) classification system for the evaluation of overall survival in gastric cancer patients: findings of a case-control analysis involving a single institution in China. Surgery.

[11] Chen HN, Chen XZ, Zhang WH (2015). Necessity of harvesting at least 25 lymph nodes in patients with stage N2–N3 resectable gastric cancer: a 10-year, single-institution cohort study. Medicine (Baltimore).

[12] Sano T, Coit DG, Kim HH (2017). Proposal of a new stage grouping of gastric cancer for TNM classification: International Gastric Cancer Association staging project. Gastric Cancer.

[13] Wang P, Deng J, Sun Z (2020). Proposal of a novel subclassification of pN3b for improvement the prognostic discrimination ability of gastric cancer patients. Eur J Surg Oncol.

[14] Zhang Z, Huang JY, Wang PL (2019). Should all stage N3b patients with advanced gastric cancer be considered equivalent? A 30-year single center study. J Gastrointest Surg.

[15] NCCN Practice Guidelines in Oncology (Gastric Cancer)-V4.2019. www.nccn.org)

[16] Cai Z, Song H, Fingerhut A (2021). A greater lymph node yield is required during pathological examination in microsatellite instability-high gastric cancer. BMC Cancer.

[17] Zhang N, Bai H, Deng J (2020). Impact of examined lymph node count on staging and long-term survival of patients with node-negative stage III gastric cancer: a retrospective study using a Chinese multi-institutional registry with Surveillance, Epidemiology, and End Results (SEER) data validation. Ann Transl Med.

[18] Smith DD, Schwarz RR, Schwarz RE (2005). Impact of total lymph node count on staging and survival after gastrectomy for gastric cancer: data from a large US-population database. J Clin Oncol.

[19] Wang J, Dang P, Raut CP (2012). Comparison of a lymph node ratio-based staging system with the 7th AJCC system for gastric cancer: analysis of 18,043 patients from the SEER database. Ann Surg.

[20] Little RJ, Rubin DB (2000). Causal effects in clinical and epidemiological studies via potential outcomes: concepts and analytical approaches. Annu Rev Public Health.

[21] Mokdad AA, Yopp AC, Polanco PM (2018). Adjuvant chemotherapy vs postoperative observation following preoperative chemoradiotherapy and resection in gastroesophageal cancer: a propensity score-matched analysis. JAMA Oncol.

[22] Schemper M, Smith TL (1996). A note on quantifying follow-up in studies of failure time. Control Clin Trials.

[23] Camp RL, Dolled-Filhart M, Rimm DL (2004). X-tile: a new bio-informatics tool for biomarker assessment and outcome-based cut-point optimization. Clin Cancer Res.

[24] Durrleman S, Simon R (1989). Flexible regression models with cubic splines. Stat Med.

[25] Nitsche U, Maak M, Schuster T (2011). Prediction of prognosis is not improved by the seventh and latest edition of the TNM classification for colorectal cancer in a single-center collective. Ann Surg.

[26] Marrelli D, Morgagni P, de Manzoni G (2012). Prognostic value of the 7th AJCC/UICC TNM classification of noncardia gastric cancer: analysis of a large series from specialized Western centers. Ann Surg.

[27] Wang W, Sun Z, Deng JY (2018). A novel nomogram individually predicting disease-specific survival after D2 gastrectomy for advanced gastric cancer. Cancer Commun (Lond).

[28] Kano K, Yamada T, Yamamoto K (2021). Evaluation of lymph node staging systems as independent prognosticators in remnant gastric cancer patients with an insufficient number of harvested lymph nodes. Ann Surg Oncol.

[29] Xu Z, Jing J, Ma G (2020). Development and validation of prognostic nomogram based on log odds of positive lymph nodes for patients with gastric signet ring cell carcinoma. Chin J Cancer Res.

[30] Park J, Jeon CH, Kim SJ (2021). A novel approach for gastric cancer staging in elderly patients based on the lymph node ratio. J Gastric Cancer.

[31] Díaz Del Arco C, Estrada Muñoz L, Sánchez Pernaute A (2021). Towards standardization of lymph-node ratio classifications: validation and comparison of different lymph node ratio classifications for predicting prognosis of patients with resected gastric cancer. Ann Diagn Pathol.

[32] Dong Y, Qiu Y, Deng J (2020). Insufficient examined lymph node count underestimates staging in pN3a patients after curative gastrectomy: a multicenter study with external validation. J Cancer Res Clin Oncol.

[33] Zhao R-S, Liu Y-N, Dai W-G (2021). A substage increase in the AJCC classification system improves prognostic prediction in stage III gastric cancer with insufficient lymph nodes removed. Front Oncol.

